# SSR marker variations in Brassica species provide insight into the origin and evolution of *Brassica* amphidiploids

**DOI:** 10.1186/s41065-017-0041-5

**Published:** 2017-07-18

**Authors:** Ajay Kumar Thakur, Kunwar Harendra Singh, Lal Singh, Joghee Nanjundan, Yasin Jeshima Khan, Dhiraj Singh

**Affiliations:** 1 ICAR-Directorate of Rapeseed-Mustard Research, Bharatpur, Rajasthan 321 303 India; 2 ICAR-Indian Agricultural Research Institute, Regional Station, Wellington, The Nilgiris, Tamilnadu 643 231 India; 3 Division of Genomic Resources, ICAR-NBPGR, PUSA Campus, New Delhi, 110 012 India

**Keywords:** *Brassica species*, Cross-transferability, Evolution, Genetic diversity, Genomic-SSRs, Phylogenetic analysis

## Abstract

**Background:**

Oilseed Brassica represents an important group of oilseed crops with a long history of evolution and cultivation. To understand the origin and evolution of Brassica amphidiploids, simple sequence repeat (SSR) markers were used to unravel genetic variations in three diploids and three amphidiploid Brassica species of U’s triangle along with *Eruca sativa* as an outlier.

**Results:**

Of 124 Brassica-derived SSR loci assayed, 100% cross-transferability was obtained for *B. juncea* and three subspecies of *B. rapa*, while lowest cross-transferability (91.93%) was obtained for *Eruca sativa*. The average % age of cross-transferability across all the seven species was 98.15%. The number of alleles detected at each locus ranged from one to six with an average of 3.41 alleles per primer pair. Neighbor-Joining-based dendrogram divided all the 40 accessions into two main groups composed of *B. juncea*/*B. nigra/B. rapa* and *B. carinata/B. napus/B. oleracea*. C-genome of oilseed *Brassica species* remained relatively more conserved than A- and B-genome. A- genome present in *B. juncea* and *B. napus* seems distinct from each other and hence provides great opportunity for generating diversity through synthesizing amphidiploids from different sources of A- genome. *B. juncea* had least intra-specific distance indicating narrow genetic base. *B. rapa* appears to be more primitive species from which other two diploid species might have evolved.

**Conclusion:**

The SSR marker set developed in this study will assist in DNA fingerprinting of various Brassica species cultivars, evaluating the genetic diversity in Brassica germplasm, genome mapping and construction of linkage maps, gene tagging and various other genomics-related studies in Brassica species. Further, the evolutionary relationship established among various Brassica species would assist in formulating suitable breeding strategies for widening the genetic base of Brassica amphidiploids by exploiting the genetic diversity present in diploid progenitor gene pools.

**Electronic supplementary material:**

The online version of this article (doi:10.1186/s41065-017-0041-5) contains supplementary material, which is available to authorized users.

## Background

**Fig. 1:**
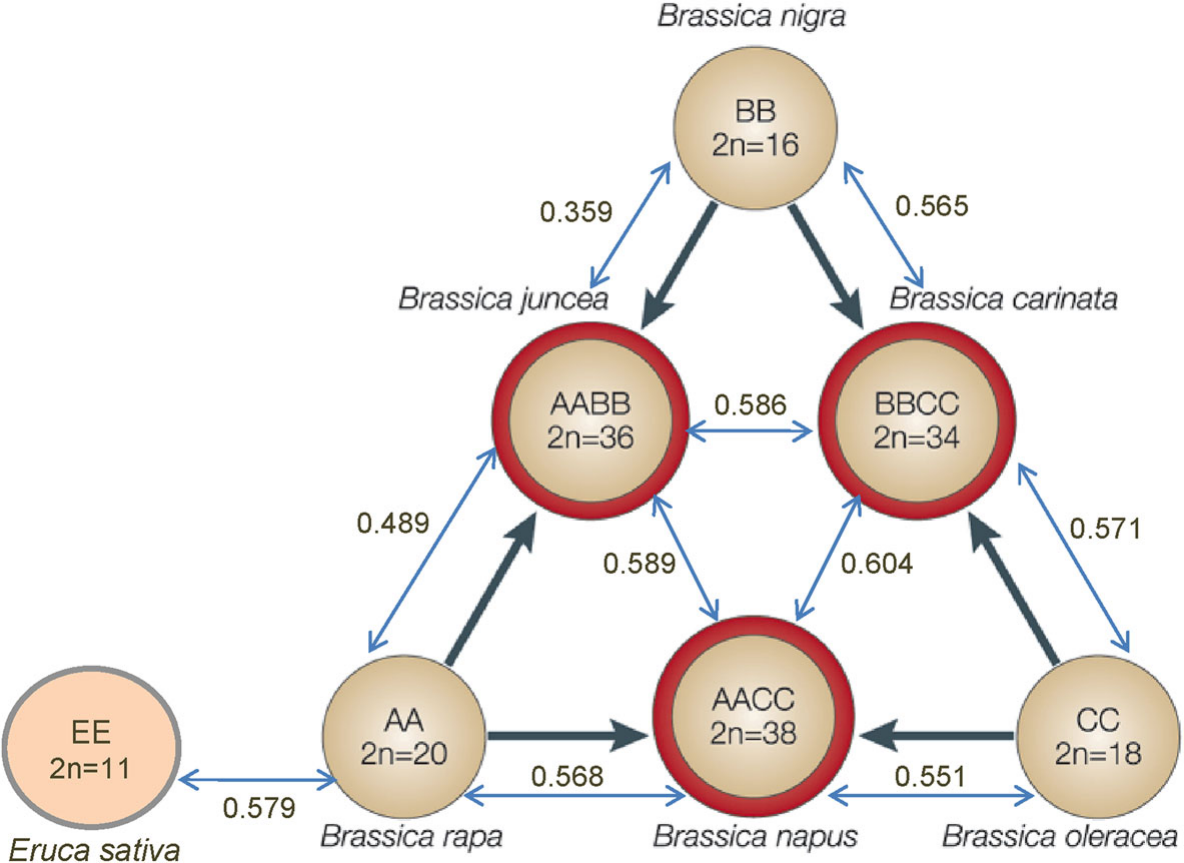
U’s triangle showing genetic relationships between Brassica diploids and amphidiploids and the genetic distances based on SSR data

Polyploidy is a widespread phenomenon among higher plants and is one of the major factors contributing to the structure and evolution of many crop species including Brassicas. Polyploidy is a natural hybridization process. The role played by hybridization had been debated for over a century and recent molecular genetic studies indicate that hybridization is amazingly occurring in natural population at a high frequency [1]. The reunion of genomes through hybridization and allopolyploidy has been estimated to account for 2–4% of speciation events in various flowering plants. Genus Brassica has a very long taxonomic and evolutionary history. It consists of three diploid species viz. *B. rapa* (2n =20, AA genome), *B. nigra* (2n = 16, BB genome), *B. oleracea* (2n = 18, CC genome) and three amphidiploid species viz. *B. juncea* (2n = 36, AABB genome), *B. carinata* (2n = 34, BBCC genome) and *B. napus* (2n = 38, AACC genome), all of which are cultivated forms, five are important oilseed crops, while *B. oleracea* is used as leafy vegetable. Among them, *B. juncea* (Indian mustard) represents the most common and widely cultivated oilseed crop, occupying >80% of rapeseed-mustard acreage in India. The relationship between the six major cultivated Brassica species was originally described by U [2], who associated the diploid Brassica species including *B. rapa*, *B. nigra* and *B. oleracea* with the amphidiploid *B. juncea*, *B. carinata* and *B. napus* (Fig. 1). *B. juncea* (AABB) is an amphidiploid containing diploid genomes from *B. rapa* (AA) and *B. nigra* (BB) [3]. *B. napus* (AACC) is a recent allotetraploid species, obtained as a result of spontaneous hybridization between the diploid species *B. rapa* (AA) and *B. oleracea* (CC). *B. napus* is an oilseed crop in many countries of Europe, Canada and Australia, and is used in industry as lubricant and biodiesel. *B. carinata* had been obtained by hybridization between *B. nigra* (BB) and *B. oleracea* (CC) and is cultivated in African countries. Since their parental genome species were thought to exist in diploid form and in different hemispheres, it became a hot research topic to explore the true progenitors for amphidiploid *Brassica species*. Further, it is also imperative to find out how their genome got modified during the course of evolutionary process.

To date, the molecular mechanism explaining the origin and evolution of rapeseed-mustard remains largely ambiguous. It is a pre-requisite to dissect the genetic relationships between these diploid and amphidiploids Brassica species. With the arrival of array of DNA-based markers, an impetus has been gained for more precise breeding, now known as molecular breeding, genetic diversity studies, phylogenetic analysis and to various crop improvement programmes [4]. However, except for *B. oleracea* and *B. rapa*, whose complete genomes have been sequenced, a very little genomic information is available for other members of *Brassicaceae* family, particularly for *B. juncea*, which is a very important oilseed crop in India, in which continuous efforts are being focused to improve several traits by exploring more number of markers, especially SSRs. The genomic evolution of Brassica allopolyploids (Chinese cultivars) had earlier been elucidated using ISSR markers [5]. However, due to lack of reproducibility and dominant nature of ISSR markers, SSR markers are the most preferred to study evolutionary process and genetic relationships. There are a number of advantages of using SSRs as they are co-dominant and multi-allelic in nature [6], offer less expensive PCR-based assay, scorability and high resolvability, and reproducibility, making them as an excellent marker system for determining phylogenetic relationships among closely related taxa.

It has been found that the genetic material and its arrangement are highly conserved among closely related species and sequence homology is found among the SSR loci flanking regions of related species [7]. Due to the conserved nature of flanking sequences, SSR markers developed in one species can be employed to detect these microsatellite loci in other related species. Among *Brassica* species including *B. rapa* (AA), *B. nigra* (BB), *B. napus* (AACC) and *B. oleracea* (CC), large number of SSR markers have been developed and many of these markers have shown to be applicable within and between different *Brassica* species. In this study, we evaluated the variation in the patterns of Brassica-derived SSR marker amplification in terms of their cross-transferability and allelic variation across six Brassica species and one related genera, *Eruca sativa*, and inferred the origin and evolutionary history of Brassica amphidiploids. This work will demonstrate the feasibility of SSR markers in resolving phylogenetic relationships of Brassica species and elucidate the possible donor species of extant Brassica amphidiploids and will also unravel the genomic changes that have taken place during the process of evolution after the formation of amphidiploids. Further the large number of SSR markers, which are reported in this study showing cross-transferability and which can reveal the intra species variability, may be much useful in diversity analysis, making heterotic pools, gene tagging etc. in cultivated species like *B. juncea*, where the genomic resources are very meager to carry out such studies.

## Methods

### Plant materials

Forty genotypes including 36 belonging to six Brassica species (3 amphidiploids; *B. juncea*, *B. carinata* and *B. napus*, and 3 diploids including *B. nigra*, *B. rapa* and *B. oleracea*) and 4 of *Eruca sativa* as an outlier, were used in the present study. *Eruca sativa* genotypes were included because of its known distant relationship to the *Brassica species* complex. The details about their ploidy level and genomic constitution are mentioned in Table 1. Actively growing shoot and leaf samples from all the forty genotypes were harvested and stored at −80 °C in the deep freezer.

**Table 1:** List of *Brassica* and related species used in the present study

Sr. no.	Species	Genotype/cultivar	Ploidy level	Genome constitution
1.	*B. nigra*	IC 262892	2×	BB
		IC 560697	2×	BB
		IC 560702	2×	BB
		IC 560713	2×	BB
2.	*B. juncea*	DRMRIJ 31	4×	AABB
		Rohini	4×	AABB
		NRCHB 101	4×	AABB
		NRCDR 2	4×	AABB
		Kranti	4×	AABB
		PusaBold	4×	AABB
3.	*B. rapa* ssp. *toria*	Agrani	2×	AA
		Bhawani	2×	AA
		Parwati	2×	AA
		PT 303	2×	AA
	ssp. *Yellow Sarson*	B 9	2×	AA
		NDYS 2	2×	AA
		NRCYS 05–02	2×	AA
		Ragini	2×	AA
	ssp. *Brown sarson*	KBS 3	2×	AA
		KOS 1	2×	AA
4.	*B. napus*	GSL 1	4×	AACC
		GSC 5	4×	AACC
		GSL 2	4×	AACC
		Sheetal	4×	AACC
		Neelam	4×	AACC
5.	*B. carinata*	Kiran	4×	BBCC
		JTC 1	4×	BBCC
		PC 5	4×	BBCC
		Pusa Swarnim	4×	BBCC
		Pusa Aditya	4×	BBCC
6.	*Eruca sativa*	T 27	2×	EE
		RTM 314	2×	EE
		TMLC 2	2×	EE
		RTM 2002	2×	EE
7.	*B. oleracea* L. var. *botrytis*	NSC cauliflower	2×	CC
		Againi	2×	CC
		Holland Special	2×	CC
		Snowball	2×	CC
	var. *capitata*	NSC cabbage	2×	CC
		Indo-American Hybrid	2×	CC

### Genomic-DNA extraction, purification and quantification

Genomic-DNA from fresh and young leaves was isolated and purified using the already standardized protocol in our laboratory [8]. The quality of the extracted DNA was evaluated by determination of A_260_/A_280_ absorbance ratio by spectrophotometer (UV-Visible Elico spectrophotometer). DNA concentration and purity were estimated by 0.8% agarose gel electrophoresis. A portion of DNA was diluted in molecular grade water to a concentration of 10 ng/μl and stored at −20 °C.

### Microsatellite markers and PCR analysis

SSR markers (124 primer pairs) derived from *B. nigra*, *B. rapa, B. napus* and *B. oleracea* were custom synthesized. Primer sequences for majority of SSR markers were obtained from http://www.brassica.info and Xu et al. [9]. *B. nigra* (BB-genome) specific SSRs were provided by Dr. S.S. Banga, National Professor (Plant Breeding & Genetics), ICAR, PAU, Ludhiana, Punjab through personal communication.

For SSR genotyping, the genomic DNA was amplified in a 25 μl reaction volume containing 50 ng DNA, 1X PCR buffer, 0.2 mM of each dNTP, 2.0 mM Mgcl_2_, 1.0 U *Taq polymerase* (MBI Fermentas, USA) and 400 nM primer using a thermal cycler (Verity 96-w Thermal Cycler, ABI, USA). The first amplification cycle consisted of initial denaturation at 94 °C for 5 min followed by 45 cycles each of denaturation at 94 °C for 30 s, primer annealing at 55 °C - 60 °C (varying with primer pair) for 30 s, primer extension at 72 °C for 45 s and a final extension step at 72 °C for 7 min. The annealing temperature (T_a_) was kept 2–3 °C below the melting temperature (T_m_) of that particular primer sequence. PCR amplified products were electrophoretically separated on 3.5% MetaPhor agarose gel containing 0.01% ethidium bromide-, prepared in 1xTAE (Tris-Acetic acid-EDTA) using 50 bp DNA ladder (Thermo Scientific, USA) as a standard reference. After electrophoresis, the amplification products were visualized in a gel documentation system fitted with 8 b CCD camera and UV light (Syngene Gel Doc, Syngene, Synoptic Ltd., UK). At least, two independent PCR amplifications were performed for each marker.

### Data analysis

Cross-transferability was determined from the presence or absence of bands on agarose gels. Each sample was assigned a ‘1’ if a band or bands was present and a ‘0’ for no band. PCR amplicons were classified into four groups on the basis of signal intensity as earlier described [10, 11]; a) strong intensity and easily scorable, b) weaker intensity and scorable, c) very week intensity and difficult to score, and d) no signal at all. Amplicons belonging to classes a) and b) were considered for positive amplification, while those belonging to classes c) and d) were considered negative for amplification and thus ignored. To determine positive amplification of a SSR marker in a species, atleast 75% of the genotypes of that species must show amplification. Cross-transferability of all the SSR markers in a species was calculated as the percentage of amplified SSRs in that species. The number of total alleles detected in all the Brassica species under study were determined for each SSR locus. The polymorphic information content (PIC) value of each SSR marker was calculated [12] using the formula; PIC = 1- Σ(P*i*)^2^, where P*i* is the frequency of the *i*th allele calculated for each SSR marker.

Further, data were scored based on the presence or absence of bands, generating a binary data matrix of 1 and 0 for each marker system and were analyzed using the DARwin 5 software [13]. The data matrices were used to calculate genetic similarity based on Jaccard’s similarity coefficient [14] and dendrogram displaying relationships among 40 genotypes was constructed by Neighbor-Joining method [15]. Intra and interspecies distances were estimated as mean of distances between n (n-1)/2 and n_1_ x n_2_ genotypes respectively, where n is the number of genotypes in a species.

## Results and discussion

### SSR marker variability/transferability across Brassica species

**Fig. 2:**
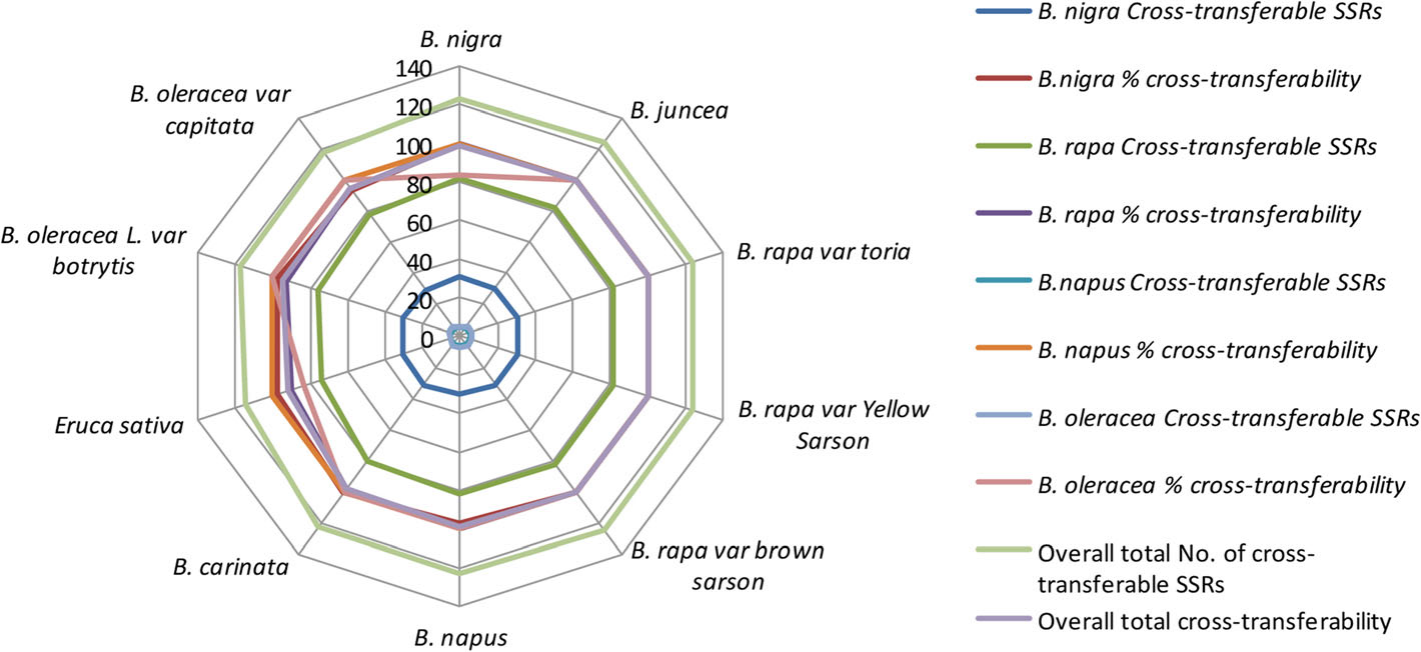
Number of cross-transferable Brassica species-derived SSR markers and % cross transferability in different species of *Brassicaceae*

Of the 124 Brassica-derived SSR loci assayed, 100% cross-transferability had been obtained for *B. juncea* and all three subspecies of *B. rapa*, while the lowest cross-transferability (91.93%) was obtained for *Eruca sativa*, where 114 SSRs showed successful amplification (Fig. 2, Additional file 1: Table S1). Out of 124 SSR markers evaluated, 114 SSRs were found to be cross-transferable across all the species under study, which infers that those SSR sites are already present / conserved in all the genotypes, indicating genome similarity and close relationship among these species. A total of 107 SSRs resulted into polymorphic amplicons. The average % age of cross amplification across all the seven species was 98.15%. The number of alleles detected at each locus ranged from one to six with an average of 3.41 alleles per primer, with a size range of 50–500 bp, which reflects huge variation among repeat regions of different alleles (Table 2). The polymorphic information content (PIC) value ranged from 0.04 (SJ1536 & BrgMS432) to 0.81 (Ol10B11 & BrgMS338). The highest average number of alleles was obtained in *B. napus* (1.91), while the lowest average number of alleles per primer pair (1.47) was found in *B. rapa* subspecies yellow sarson (Additional file 2: Table S2). A higher rate of polymorphism had been detected at inter-specific level than at intra-specific level.

**Table 2:** Cross-amplification of *Brassica*-derived SSR markers across seven species of *Brassicaceae* family

Sr.no.	Marker ID	Number of alleles										PIC value
		*B. nigra*	*B. juncea*	*B. rapa*			*B. napus*	*B. carinata*	*Eruca sativa*	*B. oleracea* L.		
				ssp. *toria*	ssp. *Yellow Sarson*	ssp. *Brown sarson*				var. *botrytis*	var. *capitata*	
A) *B. nigra*-derived SSRs												
1	SB1937	1	1	2	3	2	3	1	1	3	3	0.54
2	SJ3627R	1	1	2	2	2	2	2	1	2	4	0.72
3	SA0306	2	3	3	3	3	3	3	2	2	3	0.62
4	SJ84165	1	1	1	2	2	3	1	3	2	3	0.64
5	SJ03104	2	1	1	1	1	1	2	2	1	1	0.57
6	SJ6842	2	1	2	3	2	4	2	3	2	2	0.75
7	SJ1536	1	1	1	1	1	1	2	1	1	1	0.04
8	SB0372	1	1	2	2	2	2	1	2	2	2	0.47
9	SB31138	1	1	3	1	1	2	2	2	2	2	0.45
10	SJ3874I	2	2	2	1	2	1	1	1	1	1	0.37
11	SJ34121	2	1	1	2	1	2	3	2	2	2	0.46
12	SB3140	2	2	2	2	2	2	1	2	2	2	0.56
13	SJ3640I	1	1	1	1	2	2	2	2	1	1	0.23
14	SJ6846	2	1	2	1	1	0	1	0	0	0	0.7
15	SJ8033	4	2	2	3	2	3	3	2	3	3	0.76
16	SJ0338	2	1	2	2	3	2	2	3	2	3	0.67
17	SB2141AI	1	1	1	1	1	1	2	1	1	1	0.16
18	SJ7079	2	1	1	1	1	1	1	2	1	0	0.39
19	SJ39119I	2	1	3	3	3	3	1	2	4	3	0.65
20	SB0202I	3	3	2	2	2	2	2	2	2	2	0.61
21	SJ3627R	1	1	1	1	1	2	2	1	2	4	0.71
22	SB2131	2	1	1	1	2	1	1	2	3	2	0.64
23	SJ4933	1	1	1	1	2	1	1	1	2	1	0.56
24	SB05631	1	1	1	1	1	1	1	1	1	1	-
25	SJ3838	2	2	1	2	2	2	2	1	1	1	0.47
26	SJ3302RI	1	1	1	1	1	1	1	1	1	1	-
27	Ni4F09	2	2	1	1	2	2	2	2	1	1	0.50
28	Ni2A12	2	1	1	1	1	1	1	2	1	1	0.53
29	Ni2A02	2	1	1	1	1	1	3	1	1	1	0.40
30	Ni3C05	1	3	2	3	4	1	3	4	2	2	0.74
31	Ni2D10	1	1	1	1	1	1	1	1	1	1	-
B) *B. rapa*-derived SSRs												
32	BrgMS418	2	2	2	1	1	3	3	2	3	3	0.72
33	BrgMS68	2	1	1	1	2	1	1	2	1	2	0.32
34	BrgMS75	1	1	2	2	2	2	4	3	4	3	0.64
35	BrgMS804	1	1	1	1	1	3	2	1	1	1	0.34
36	BrgMS422	1	1	3	2	2	1	2	1	2	1	0.47
37	BrgMS426	1	1	2	1	3	1	1	1	1	1	0.14
38	BrgMS135	2	1	2	2	2	3	2	2	2	2	0.46
39	BrgMS1237	1	1	2	1	2	2	1	1	1	1	0.62
40	BrgMS801	1	1	1	1	1	1	1	2	1	2	0.45
41	BrgMS397	1	1	3	1	2	2	2	0	3	1	0.49
42	BrgMS521	1	1	1	1	1	1	1	1	1	1	0.25
43	BrgMS745	1	1	1	1	1	1	2	2	2	3	0.35
44	BrgMS432	1	1	2	1	1	1	1	1	1	1	0.04
45	BrgMS792	2	2	2	2	2	2	2	2	2	2	0.5
46	BrgMS334	3	3	3	3	3	3	3	3	3	3	0.67
47	BrgMS316	5	3	1	1	1	2	2	4	1	1	0.72
48	BrGMS4497	3	3	2	2	2	5	6	1	4	5	0.76
49	BrgMS372	2	2	2	1	3	3	1	3	1	1	0.65
50	BrgMS2766	1	1	1	1	2	1	2	1	1	2	0.61
51	BrgMS751	1	1	1	1	1	1	1	1	1	1	-
52	BrgMS413	1	1	1	1	1	1	1	1	1	1	-
53	BrgMS313	1	1	1	1	1	1	1	1	1	1	-
54	BrgMS359	2	1	1	2	1	2	1	1	2	2	0.39
55	BrgMS793	1	1	1	1	1	1	1	1	1	1	-
56	BrgMS430	1	1	2	1	2	1	1	1	1	1	0.33
57	BrgMS710	1	1	1	1	1	1	1	1	1	1	-
58	BrgMS1238	1	2	2	1	2	2	2	1	1	1	0.46
59	BrgMS412	1	1	2	1	3	2	1	1	2	1	0.41
60	BrgMS722	1	1	1	1	1	1	1	1	1	1	-
61	BrgMS787	2	2	2	3	3	2	2	1	1	2	0.66
62	BrgMS399	1	1	1	1	1	2	1	1	2	1	0.23
63	BrgMS713	2	2	3	2	4	4	2	1	2	1	0.71
64	BrgMS4508	2	1	4	2	2	3	2	1	3	2	0.72
65	BrgMS4533	3	1	2	1	2	2	2	2	1	2	0.58
66	BrgMS4536	2	2	2	2	2	2	1	1	1	1	0.46
67	BrgMS63	1	1	1	1	1	1	1	1	1	1	0.09
68	BrgMS490	1	1	1	1	2	2	1	4	1	3	0.59
69	BrgMS776	1	1	1	1	2	3	3	1	1	2	0.37
70	BrgMS961	1	1	1	1	3	2	1	1	2	2	0.52
71	BrgMS794	2	2	1	1	1	2	1	1	2	3	0.69
72	BrgMS724	1	1	2	2	2	3	1	1	1	1	0.31
73	BrgMS337	2	3	1	1	2	1	1	0	0	1	0.57
74	BrgMS70	4	3	2	1	2	2	0	0	0	0	0.71
75	BrgMS2767	2	2	2	2	2	1	1	2	2	2	0.42
76	BrgMS778	1	1	1	1	1	1	1	1	1	1	0.18
77	BrgMS802	2	1	2	2	1	2	1	1	1	2	0.62
78	BrgMS344	1	1	1	1	2	1	1	1	2	2	0.64
79	BrgMS783	1	1	1	1	2	3	1	1	1	1	0.52
80	BrgMS780	2	1	1	1	1	1	2	1	1	1	0.25
81	BrgMS350	1	1	1	1	1	1	1	1	0	1	-
82	BrgMS388	2	1	2	1	2	4	2	1	4	3	0.69
83	BrgMS60	1	1	1	1	1	2	2	1	1	1	0.14
84	BrgMS516	1	1	1	1	2	2	1	1	1	1	0.16
85	BrgMS777	3	3	3	2	2	3	3	2	2	2	0.57
86	BrgMS309	2	1	1	1	1	1	1	0	0	0	0.12
87	BrgMS338	3	4	3	2	2	4	2	0	2	2	0.81
88	BrgMS782	1	3	1	1	1	1	1	1	1	3	0.64
89	BrgMS457	1	3	3	3	2	2	3	2	1	1	0.71
90	BrgMS710	3	1	2	1	1	2	2	1	1	3	0.56
91	BrgMS732	4	3	2	1	4	3	3	3	3	2	0.68
92	BrgMS372	2	2	2	1	3	3	1	3	1	1	0.65
93	BrgMS339	2	1	1	2	4	2	1	0	2	1	0.71
94	BrgMS409	2	1	1	1	1	2	2	1	1	2	0.32
95	BrgMS753	1	1	1	1	1	1	3	2	3	4	0.55
96	BrgMS421	3	2	2	1	2	2	1	2	2	2	0.56
97	BrgMS799	2	2	2	2	3	2	1	2	1	1	0.54
98	BrgMS343	1	1	1	1	1	1	2	1	2	2	0.35
99	BrgMS638	1	1	2	3	2	2	2	2	2	2	0.60
100	BrgMS515	1	1	1	1	1	1	1	2	1	1	0.05
101	BrgMS354	1	1	1	1	1	1	1	1	1	1	-
102	BrgMS777	1	1	1	1	1	1	1	1	1	1	-
103	BRMS-002	2	2	2	2	2	2	2	2	2	2	0.56
104	BRMS-016	2	1	1	1	1	2	3	2	2	2	0.67
105	BRMS-011	1	1	2	4	3	2	0	0	0	0	0.72
106	BRMS-005	2	1	2	1	3	2	1	1	1	1	0.65
107	BRMS-003	1	1	1	1	2	2	1	1	1	1	0.47
108	BRMS-029	1	2	1	1	2	2	1	2	1	1	0.48
109	BRMS-007	2	3	1	1	2	3	1	1	1	1	0.78
110	BRMS-015	2	2	2	1	2	2	2	1	3	2	0.49
111	BRMS-006	4	2	3	1	2	4	2	0	0	0	0.75
112	BRMS-014	1	1	1	1	1	1	1	1	1	1	-
113	BRMS-033	1	1	1	1	1	1	1	1	1	1	-
C) *B. napus*-derived SSRs												
114	MB4	2	2	1	1	1	1	2	1	1	1	0.47
115	MR52a	2	1	2	1	1	3	2	1	2	2	0.53
116	MR33	1	1	1	1	1	1	1	1	1	1	-
117	MR176	2	5	4	2	3	3	2	1	3	2	0.77
118	MB5	1	1	1	1	1	1	1	1	1	1	-
D) *B. oleracea*-derived SSRs												
119	sORA43	1	3	2	4	3	2	2	1	1	3	0.68
120	Ol09A01	1	1	1	1	1	1	1	1	1	1	-
121	Ol10B01	2	2	2	4	3	3	1	1	2	2	0.73
122	Ol10B07	0	1	1	2	1	3	3	0	3	5	0.79
123	Ol10B11	3	3	3	3	2	3	3	1	1	2	0.81
124	sORA26	2	2	2	2	2	3	1	3	1	2	0.59

The overall frequency of cross species-SSR marker transferability (average 98.15%) in the present investigation is much higher than that observed in earlier studies among various *Brassica species* and related genera [16]; where they could obtain a cross species-transferability frequency of 62.3% and 71.7%, respectively. Recently, comparative genomics in Brassica have shown that microsatellite characteristics in related species are highly similar [17]. Intra-generic transferability of SSRs had been reported earlier in many studies, eg. SSRs from *Pennisetum glaucum to P. purpureum* [18], *Brassica species* to *B. tournefortii, B. fruticulosa* and *B. spinescens* [19]. However, transferability of SSR markers to distantly related species has also been reported [20]. Transferability and polymorphic potential of various Brassica-derived SSR markers among Brassica species had also been investigated earlier [21, 22]. In another study, transferability of SSR markers between A- and C- genomes of Brassica species had been evaluated [23], which corresponded to the already established evolutionary relationship. However, we are reporting here a new set of SSR markers in addition to the already validated SSRs in different genotypes of Brassica species, which would be helpful in Brassica genomics studies, particularly for *B. juncea,* where very little genomic information is available.

### Phylogenetic relationship between diploid and amphidiploids species

**Fig. 3:**
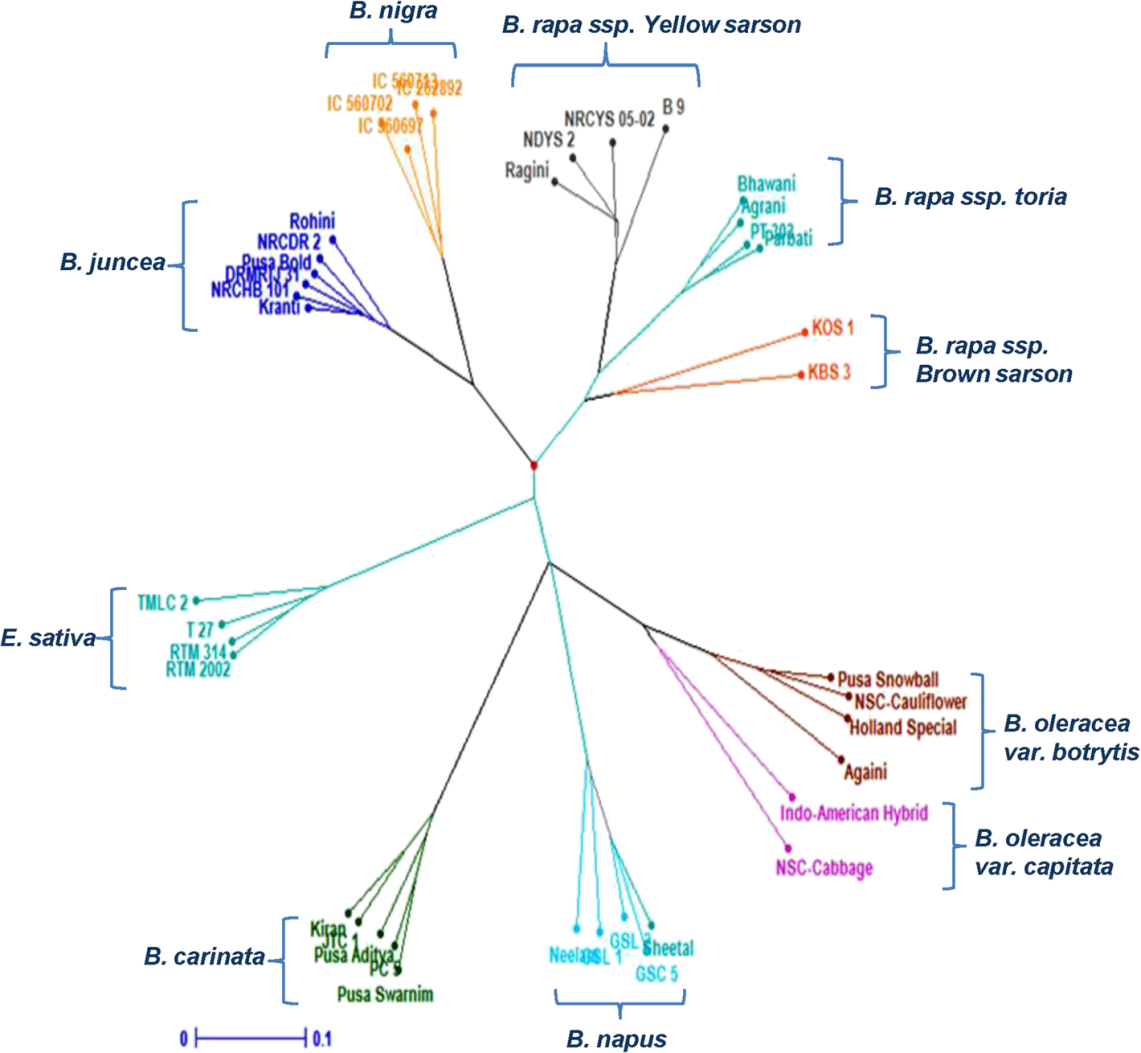
Unweighted Neighbor-Joining (UNJ) dendrogram depicting the genetic relationship among different species of *Brassicaceae* based on allelic data of SSR markers

In order to establish a clear basis for establishing the origin of Brassica ampidiploids, we used SSR markers to provide baseline evidence to clarify the possible origins of various species. The NJ-based dendrogram divided all the 40 accessions into two main groups, respectively composed of *B. juncea*/*B. nigra/B. rapa* (ssp. yellow sarson, toria and brown sarson) i.e. AB- genome, B- and A- genome species; and *B. carinata/B. napus/B. oleracea var. capitata* and *botrytis* i.e. BC-, AC- and C- genome species (Fig. 3). Species comprising of A- and B- genome has fallen in different groups, while all the species having C genome grouped together. It clearly demonstrates that A- and B- genomes of oilseed *Brassica species* have undergone more genomic changes than C- genome after amphidiploidization and intensive cultivation. Though *B. oleracea*, a vegetable species has worldwide distribution, yet C- genome present in oilseed Brassica species *B. carinata* and *B. napus* remained conserved, hence there is a need to introgress C- genome from different sources to create more diversity in these species. Grouping of *B. juncea* (AABB) and *B. napus* (AACC) into two different groups in the present study is an indication of diversity between A- genome of both species. A recent study reported that A- genome of *B. juncea* and *B. napus* each had independent origins [24] and this information may shed light on the unusual features of selection divergence in Brassica. Thus introgression of individual A-genome types may be carried out to synthesize Brassica amphidiploids to achieve more diversity for breeding objectives.

Intra-species distance was highest in *B. oleracea* followed by *B. rapa* (Table 3). Both species comprise of subspecies indicating much variability within these species. Least intra-species distance was observed in *B. juncea* depicting its narrow genetic base. *B. oleracea* and *B. rapa* are grown worldwide over wide geographic ranges, therefore have accumulated more diversity; while *B. juncea* is mostly restricted to south Asia and that is the reason behind its narrow genetic variability [25]. Interspecies distances throw light on their contribution in evolutionary process. Highest interspecies distance was found between *B. carinata* (BBCC) and *E. sativa* (EE) indicating relatively low exchange of genetic material between these two species, hence these are the most unrelated ancestors. It was quite interesting to note that among three amphidiploids, *B. carinata* and *B. napus* had highest genetic distance with *E. sativa,* while *B. juncea* had more distance with *B. oleracea* than *E. sativa*. It indicates the possibility of exchanging genetic material between *B. juncea* and *E. sativa* more frequently than between *B. oleracea/B. napus* and *E. sativa*.

**Table 3:** Genetic distances between different species of *Brassicacaeae* under present investigation

	*B. nigra*	*B. juncea*	*B. rapa*			*B. napus*	*B. carinata*	*Eruca sativa*	*B. oleracea*	
			*ssp*. *toria*	*ssp. yellow sarson*	*ssp. brown sarson*				var. *botrytis*	var. *capitata*
*B. nigra*	0.191									
*B. juncea*	0.359	0.132								
*B. rapa ssp. toria*	0.539	0.488	0.133							
*B. rapa ssp. yellow sarson*	0.558	0.481	0.338	0.171						
*B. rapa ssp. brown sarson*	0.556	0.509	0.363	0.429	0.351					
*B. napus*	0.607	0.589	0.567	0.577	0.553	0.206				
*B. carinata*	0.586	0.583	0.579	0.569	0.599	0.622	0.184			
*Eruca sativa*	0.585	0.583	0.579	0.569	0.599	0.622	0.693	0.184		
*B. oleracea* var. *botrytis*	0.661	0.638	0.617	0.592	0.624	0.553	0.568	0.603	0.203	
*B. oleracea* var. *capitata*	0.662	0.643	0.615	0.596	0.6164	0.545	0.574	0.592	0.387	0.347

*B. juncea* was closer to *B. nigra* (BB) than *B. rapa* (AA) which suggests exchange of genetic information between these two species through natural hybridization during evolution. It had been reported that *B. juncea* might have originated several times with both *B. rapa* and *B. nigra* as cytoplasmic donors in separate hybridization events [26]. *B. napus* had almost equal genetic distance with its ancestors *B. oleracea* (CC, 0.551) and *B. rapa* (AA, 0.568). More extensive breeding programmes in *B. napus* have resulted into introgression of both AA and CC genomes equally. Another study ruled out the possibilities of any close relationship of *B. oleracea* (CC) or any of the C-genomes species with the maternal progenitor of *B. napus* (AACC) using chloroplast and nuclear-SSR markers [27]. They also proposed that multiple hybridization events involving different maternal ancestors might have produced *B. napus*. In a previous study, a fully resolved chloroplast phylogeny of various Brassica crops and wild relatives (from USA) had been established using four plastid region based markers [28], where various Brassica species were placed in either of the two most species-rich clades i.E. *nigra* and Oleracea. They grouped *E. sativa*, *B. napus*, *B. juncea*, *B. rapa* and *B. oleracea* into Oleracea clade, while *B. nigra* and *B. carinata* were placed into Nigra clade. Highest interspecies distance between *E. sativa* and *B. carinata* in our nuclear genome based study is in conformity with the grouping of these two species in different clades based on plastid genome based study, but differs for grouping of *B. oleracea* which might have happened due to the exchange of nuclear genome between *B. carinata* and *B. oleracea* in recent breeding programmes.

Similarly *B. carinata* also has equal distances with *B. nigra* (BB, 0.564) and *B. oleracea* (CC, 0.570). Among the A-, B- and C- genome; A- was nearer to B- (0.552) than C- (0.608), while B- and C- were at farthest distance (0.661). It would have been due to different regions of their cultivation. On the basis of genetic distances between diploid species, we propose *B. rapa* as the most primitive ancestor of U’s triangle which would have undergone changes to evolve other two diploid species. Further insight was sought to look into three subtypes of *B. rapa* viz. toria, yellow sarson and brown sarson. Toria subtype was nearer to yellow sarson than brown sarson, while yellow sarson and brown sarson were at relatively more genetic distance. Among the three subtypes of *B. rapa*, toria has been derived from brown sarson through selection for earliness to fit well in the cropping systems. *B. rapa* var. brown sarson seems to be the primitive ancestor of all diploid species. Yellow sarson is characterized by yellow seed coat color, tetralocular siliquae, semi-erect plant type and self-mating system, while brown sarson encompasses two different forms lotni (cross-pollinated) and tora (self-pollinated), brown seed coat color and bilocular siliquae. Yellow sarson which is closely related with brown sarson would have evolved as mutant of brown sarson. However, the peroximity of toria with yellow sarson in the present study would have been due to winter type brown sarson varieties. A similar study revealed the phylogenetic relationships among cultivated *B. rapa ssp. rapa*, *ssp. oleifera*, *ssp. pekinensis*, *ssp. chinensis* and *ssp. japonica* using AFLP markers and concluded that *B. rapa* cultivars from east Asia were probably derived from a primitive cultivated type, which might have originated in Europe or in central Asia and then migrated to east Asia [29].

Molecular markers are excellent tools to study the genetic relationships and genomic evolution of polyploid species. Genetic relationships among Brassica species (Indian genotypes) as depicted by SSR markers in the present investigation were in agreement with the diploid/amphidiploids relationship as described by U [2]. It may be inferred that the ancient amphidiploid Brassica species were formed possibly by hybridization and chromosome doubling between archaic species related to *B. rapa*, *B. nigra*, *B. oleracea*.

## Conclusion

In conclusion, the high level of SSR marker cross-transferability observed in this study demonstrated the usefulness of various Brassica-derived SSR markers for the analysis of genetic relationship and provided insights into the genomic evolution of various diploid and amphidiploids Brassica species. This SSR marker set will assist in DNA fingerprinting of various Brassica species cultivars, evaluating the genetic diversity in Brassica germplasm, genome mapping and construction of linkage maps, gene tagging and various other genomics-related studies in Brassica species. This research investigation has attempted to find out the diploid progenitors of various Brassica amphidiploids. C-genome of oilseed *Brassica species* remained relatively more conserved than A- and B-genome. A- genome present in *B. juncea* and *B. napus* seems distinct from each other and hence provides great opportunity for generating diversity through synthesizing amphidiploids from different sources of A- genome. *B. juncea* had least intra-specific distance indicating narrow genetic base. *B. rapa* appears to be more primitive species from which other two diploid species might have evolved. Among three subtypes of *B. rapa*, brown sarson appeared the most primitive progenitor. *Eruca sativa* was found to be closer to *B. juncea* than *B. carinata* and *B. napus*. Suitable breeding strategies can be formulated for widening the genetic base of Brassica amphidiploids by exploiting the genetic diversity present in diploid progenitor gene pools (A-, B- & C-).

## Additional files

Additional file 1: Table S1. Amplification frequency of *Brassica*-derived SSR markers across seven species of *Brassicaceae* family. (DOCX 13 kb)

Additional file 2: Table S2. Allelic data of cross-transferable SSRs in the present investigation. (DOCX 12 kb)

## Abbreviations

AFLP: Amplified fragment length polymorphism
CTAB: Cetyl tri-ammonium bromide
ISSR: Inter simple sequence repeat
NJ: Neighbor-Joining
PCR: Polymerase chain reaction
RAPD: Random amplified polymorphic DNA
RFLP: Restriction fragment length polymorphism
SSR: Simple sequence repeat
