# Supplementary material for: SSR marker variations in Brassica species provide insight into the origin and evolution of Brassica amphidiploids
Source: Hereditas. 2017 Jul 18;155:6. doi: 10.1186/s41065-017-0041-5 (PMC5516320; doi:10.1186/s41065-017-0041-5)
Supplement: Supplementary file 1 — Amplification frequency of Brassica-derived SSR markers across seven species of Brassicaceae family. (DOCX 13 kb) [file 41065_2017_41_MOESM1_ESM.docx]

**Table S1 Amplification frequency of *Brassica*-derived SSR markers across seven species of *Brassicaceae* family**

| **Source of SSR markers** |  | ***B. nigra*** | ***B. juncea*** | ***B. rapa*** | | | ***B. napus*** | ***B. carinata*** | ***Eruca sativa*** | ***B. oleracea* L.** | |
| --- | --- | --- | --- | --- | --- | --- | --- | --- | --- | --- | --- |
|  |  |  |  | **ssp. *toria*** | **ssp. *Yellow Sarson*** | **ssp. *Brown sarson*** |  |  |  | **var. *botrytis*** | **var. *capitata*** |
| ***B. nigra***  Total SSRs = 31 | Cross-transferable SSRs | 31 | 31 | 31 | 31 | 31 | 30 | 31 | 30 | 30 | 29 |
|  | % cross-transferability | 100 | 100 | 100 | 100 | 100 | 96.77 | 100 | 96.77 | 96.77 | 93.54 |
| ***B. rapa***  SSRs = 82 | Cross-transferable SSRs | 82 | 82 | 82 | 82 | 82 | 82 | 80 | 74 | 76 | 78 |
|  | % cross-transferability | 100 | 100 | 100 | 100 | 100 | 100 | 97.56 | 90.24 | 92.68 | 95.12 |
| ***B. napus***  SSRs = 5 | Cross-transferable SSRs | 5 | 5 | 5 | 5 | 5 | 5 | 5 | 5 | 5 | 5 |
|  | % cross-transferability | 100 | 100 | 100 | 100 | 100 | 100 | 100 | 100 | 100 | 100 |
| ***B. oleracea***  SSRs = 6 | Cross-transferable SSRs | 5 | 6 | 6 | 6 | 6 | 6 | 6 | 5 | 6 | 6 |
|  | % cross-transferability | 83.33 | 100 | 100 | 100 | 100 | 100 | 100 | 83.33 | 100 | 100 |
| Overall total No. of cross-transferable SSRs | | **123** | **124** | **124** | **124** | **124** | **123** | **122** | **114** | **117** | **118** |
| Overall total cross-transferability | | **99.19** | **100** | **100** | **100** | **100** | **99.19** | **98.38** | **91.93** | **94.35** | **95.16** |
