# Supplementary material for: SSR marker variations in Brassica species provide insight into the origin and evolution of Brassica amphidiploids
Source: Hereditas. 2017 Jul 18;155:6. doi: 10.1186/s41065-017-0041-5 (PMC5516320; doi:10.1186/s41065-017-0041-5)
Supplement: Supplementary file 2 — Allelic data of cross-transferable SSRs in the present investigation. (DOCX 12 kb) [file 41065_2017_41_MOESM2_ESM.docx]

**Table S2 Allelic data of cross-transferable SSRs in the present investigation**

| **S. No.** | **Species/ssp.** | | **Total No. of cross-transferable SSR markers** | **Total No. of alleles** | **Total No. of monomorphic alleles** | **Total No. of polymorphic alleles** | **Average No. of alleles/marker** |
| --- | --- | --- | --- | --- | --- | --- | --- |
| 1 | *B. nigra* | | 123 | 203 | 142 | 61 | 1.65 |
| 2 | *B. juncea* | | 124 | 187 | 137 | 50 | 1.5 |
| 3 | *B. rapa* | ssp. *toria* | 124 | 199 | 138 | 61 | 1.6 |
|  |  | ssp. *Yellow Sarson* | 124 | 183 | 132 | 51 | 1.47 |
|  |  | ssp. *Brown sarson* | 124 | 219 | 148 | 71 | 1.76 |
| 4 | *B. napus* | | 123 | 235 | 151 | 84 | 1.91 |
| 5 | *B. carinata* | | 122 | 200 | 137 | 63 | 1.64 |
| 6 | *Eruca sativa* | | 114 | 173 | 123 | 50 | 1.51 |
| 7 | *B. oleracea* L. | var. *botrytis* | 117 | 188 | 127 | 61 | 1.6 |
|  |  | var. *capitata* | 118 | 204 | 139 | 65 | 1.72 |
